# Effect of oral eplerenone in anatomical and functional improvement in patients with chronic central serous chorioretinopathy

**DOI:** 10.12669/pjms.35.6.896

**Published:** 2019

**Authors:** Omer Farooq, Asad Habib, Masood Alam Shah, Najia Ahmed

**Affiliations:** 1 Dr. Omer Farooq, MBBS, FCPS, FRCS. Department of Ophthalmology, PNS Shifa Hospital, BUMDC, Karachi, Pakistan; 2 Dr. Asad Habib, MBBS. Department of Ophthalmology, PNS Shifa Hospital, BUMDC, Karachi, Pakistan; 3 Dr. Masood Alam Shah, FCPS. Department of Ophthalmology, PAF Hospital Faisal, Karachi, Pakistan; 4 Dr. Najia Ahmed, MBBS, FCPS. Department of Dermatology, PNS Shifa Hospital, BUMDC, Karachi, Pakistan

**Keywords:** Central serous retinopathy, Eplerenone, Mineralocorticoids, Subretinal fluid

## Abstract

**Objective:**

To determine the efficacy of oral eplerenone in anatomical and functional improvement in patients with chronic central serous chorioretinopathy (CSCR).

**Methods:**

This quasi experimental study was performed at PNS Shifa Hospital Karachi from September 2018 to February 2019. Study included 23 patients. Patients were included using consecutive sampling technique and informed consent was taken from all patients before staring treatment. 50 mg of oral eplerenone per day was given for three months. Subretinal fluid (SRF) height and visual acuity (VA) were noted at baseline, one month and three month follow-up. Structured Study performa was used for data collection. Data was analysed and assessed with SPSS version 23. P value of <0.05 was considered statistically significant.

**Results:**

Mean age of patients was 40.7±7 years and mean duration of disease before treatment was 3.7±0.76 months. Mean baseline BCVA and SRF height was 0.39±0.02 logMAR and 123±12.5 µm respectively. Sixty-five percent patients responded at one month and 80% at three months with reduction in SRF height. Improvement in visual acuity was also statistically significant at 3 months (p<0.05).

**Conclusion:**

Use of eplerenone in chronic CSCR resulted in significant improvement in vision and decrease in mean SRF height.

## INTRODUCTION

CSCR is a sight threatening condition associated with serous exudation between pigmentary and neurosensory retinal causing a localized retinal detachment.^1^CSCR is considered 4^th^ most common cause of medical retinopathy after age related macular degeneration (ARMD), retinal venous occlusion (RVO) and diabetic retinopathy (DR).^2^

Typically, presentation includes unilateral, painless deterioration of vision. However, in few cases it can become chronic or recurrent. Bilateral but asymmetric disease is not uncommon. Disease can go unnoticed if fovea is spared. Symptoms usually resolve within one to four months as the disease is self-limiting.^2^ Disease is considered chronic if it persists from 3 to 6 months. Patient may complaint of metamorphopsia, micropsia/macropsia or a visual field scotoma or a combination of these symptoms. 3

Many risk factors have been associated with CSCR including type A personality, smoking, hypertention, pregnancy, male gender, anxiety, stress, gastroesophagial reflux disease (GERD), *H.pylori*, both exogenous and endogenous steroids.^4^

Studies show that damage to outer retinal barrier is the prime pathology leading to subretinal exudation.^5^ Several treatment modalities have been tried including observation for spontaneous resolution, focal laser, photodynamic therapy (PDT) oranti vascular endothelial growth factor agent (anti-VEGF).^2^,^5^ Focal or micropulse laser stimulates retinal pigment epithelium (RPE) pump and helps pull out excess water while PDT with verteporfin causes long term choroidal vasculature remodelling. Anti-VEGF has also been studied as a possible treatment option. Lim et al.^6^ found no VEGF in vitreous in cases of CSCR. Hence anti-VEGF was not reported to be effective unless associated with choroidal neovascularisation (CNV). Role of mifeprisone, acetazolamide, rifampicin, finasteride, ketoconazole and anti *H.pylori* treatment has been studied by various authors with no definitive evidence in any large randomized control trial. None of these is so far accepted as a definitive treatment. Several investigators postulated role of upregulation of mineralocorticoid receptors and choroidal vascular hyperpermeablity in CSCR.^7^,^8^

Eplerenone is a competitive antagonist of mineralocorticoids and has been shown to be a new promising treatment modality for CSCR.^9^ However current literature lacks a large cohort in this perspective. Purpose of this study was to determine the efficacy of this new treatment modality, eplerenone in patients with CSCR.

## METHODS

Twenty-three patients of chronic CSCR were included in the study using the consecutive sampling technique. Chronic CSCR was defined for this study as the one in which SRF didn’t resolve after an observation period of minimum three months. Previously treated patients were excluded. Patients with other retinal pathologies like AMD, RVO, DME, uveitis were also excluded.

Hospital ethical committee approval was obtained (PNS Shifa hospital ethical review committee, dated 1-09-2018) Informed consent was taken. Data collection and reporting were in accordance with Declaration of Helsinki. Oral Eplerenone in dose of 50 mg per day was started. Study parameters were best corrected visual acuity (taken on snellen chart converted into logMAR units) and maximum SRF height on spectral domain optical coherence tomography (Spectralis SD-OCT, Heidelberg Engineering, Heidelberg Germany). These variables were recorded at baseline (before start of treatment) and at one and three months after start of treatment. Patients were observed for possible side effects and their appropriate management; however there were no drop outs from the study on this basis.

### Statistical analysis

Statistical package for social sciences (SPSS 23.0) for windows was used for statistical analysis. P-value <0.05 considered significant. Two-tailed paired t-test was used for analysis.

## RESULTS

Twenty-five eyes of 23 patients including 20 men (80%) and 5 women (20%) with a mean age of 40.7 ± 7 years were treated with eplerenone 25 mg twice daily. Two patients had bilateral chronic serous chorioretinopathy. Each eye of these two patients was considered independently. Overall fourteen eyes were right and eleven were left. Mean duration of disease before tablet eplerenone was started was 3.7±0.76 months. Mean baseline BCVA was 0.39±0.02 logMAR and mean baseline SRF height was 123±12.5 µm.

**Fig. 1:**
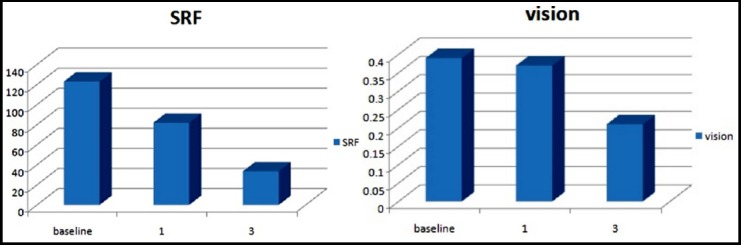
Mean vision (logMAR) and SRF height (microns) at baseline one month and 3 months.

In 65% patients, a decrease in SRF height was observed at 1 months with mean decrease of 64.5±11.6µm (p<0.05) and 80% at three months with mean decrease of 110±12.6 µm (p<0.05).In 20% patients no decrease of SRF was observed even after 3 months. At one-month follow-up mean BCVA improved to 0.37±0.02 logMAR at one month (p<0.05) and 0.21±0.15 logMAR at 3-month follow-up (p<0.05) (Fig. 1).

Out of 9 patients (36%) who didn’t document any improvement in SRF height at one month, four eyes (16% of total) responded between 1-3 month with one eye having no final residual SRF and mean residual SRF height of 37±12 µm in rest three eyes.15 of 25 eyes (60%) had no residual SRFafter3 months of treatment with eplerenone.

## DISCUSSION

Eplerenone is a mineralocorticoid receptor antagonist. It poorly binds to other receptors like androgens and thus has less adverse effects than other antagonists with good androgen sensitivity. Several researchers have tried to find pathogenesis of CSCR and many theories exist. To start with Gass floated the first theory of focal choroidal hyper-permeability followed by Marmor who proposed that it’s a functional damage rather than an anatomical one at RPE level that leads to fluid accumulation.^10^,^11^ Similarly choroidal ischemia, hypermeabililty, RPE dysfunction were studied by various researchers in the past. Recently, progress has been made in knowing the molecular basis of CSCR. Knowing that CSCR worsens with the use of steroids, and both endogenous and exogenous steroids being a risk factor of CSCR, the possible role of steroids antagonists was proposed and researched upon.^12^,^13^

These observations lead the way to evaluate role of mineralocorticoids receptor antagonists in CSCR. They were Zhao et al^14^ and Daruich et al^15^ who studied the role of glucocorticoids and mineralocorticoids and their specific receptors on choroidal vasculature. Zhao et al. reported that the effect of eplerenone lasted for five months after drug cessation in two patients of CSCR.^14^ Later many researchers investigated the efficacy and safety of eplerenone in CSCR. Most of these studies were however of short term follow-up duration and none was a randomised trial. Most studies were retrospective. Largest of these studies was by Sampo et al^16^ who included 27 patients and Gergely et al^17^ who studied 28 cases with 3 and 6 months follow-up respectively.

Rahimy et al. and Schwartz et al. are the only ones who reported superiority of eplerenone over placebo in a prospective randomized study in terms of visual acuity, central macular thickness and SRF height.^18^,^19^ However their subject size was small (n =15 and 13 respectively) and follow-up was nine weeks and six months respectively. However in cases of prolong disease or RPE damage eplerenone may not be that effective or need an earlier intervention before a widespread RPE damage.^20^ Foveal detachment, CNV, subretinal and fibrosis are other reported bad prognostic factors.

Recently Kapoor et al. did follow-up for 12 months and reported an improvement in condition but the subject size was small (n=12).^21^ These studies reported variable dose dependent reversible adverse effects including but not limited to diabetes, cardiac failure, drug interactions, fatigue, dizziness, hypokalemia, bowel disturbance, weight loss, gynecomastia and altered liver enzymes.

Chatziralli et al. recommended a dose of 25-50 mg/day of eplerenone as safe as well as effective in chronic CSCR.^22^ Bousquet advised 25 mg/day for one week and 50 mg /day for next one to three months^7^.

Rahimy et al. in one of the only two randomized trial on the use of eplerenone used a dose of 25 mg daily for one week, then up to 50 mg daily for 8 weeks. They defined chronic CSCR as the one in with at least three months of treatment with persisting symptoms and less than 50% reduction in SRF. In the second randomized trial Schwartz used dose of 50 mg/day. Both Rahimy and Schwartz advocated safe and effective role of eplerenone in chronic CSCR.

Our study showed that Eplerenone was effective in majority of patients in reducing SRF and improving VA. Still a fair percentage (20%) didn’t show any improvement even at three months. This showed that there are other known or unknown factors in the pathogenesis which caused treatment failure.

### Limitations of study

Sample size is small. But its comparable to the recent studies on the topic. The disease is a relatively a rare one and finding a large patient group on this is not possible in our circumstances. Relevant recent studies and meta-analysis in last five years had similar smaller sample sizes, (maximum was 28 by Gergely et al.) supporting my point number. Although we assessed patients for possible side effects however no patient was dropped from the study because of better safety profile of drug. A randomized trial on a large cohort with long term follow-up for adverse effects and safety profile is recommended.

## CONCLUSION

Eplerenone is effective in reducing the SRF height on OCT and improve visual acuity in cases of chronic CSCR.

### Author`s Contribution:

**OF:** Basic idea, data collection, final approval and research integrity.

**MAS:** Literature search.

**AH:** Manuscript writing.

**NA:** Statistical analysis.
